# Bone Mineral Density and Trabecular Bone Score in Predicting Vertebral Fractures in Male Employees of the Electricity Generating Authority of Thailand

**DOI:** 10.1155/2022/6832166

**Published:** 2022-03-29

**Authors:** Chaninart Sakulpisuti, Chanika Sritara, Arpakorn Kositwattanarerk, Praman Fuangfa, Chaiyawat Suppasilp, Prin Vathesatogkit, Dujrudee Matchariyakul, Boonsong Ongphiphadhanakul, Piyamitr Sritara

**Affiliations:** ^1^Division of Nuclear Medicine, Department of Diagnostic and Therapeutic Radiology, Faculty of Medicine Ramathibodi Hospital, Mahidol University, Bangkok, Thailand; ^2^Division of Diagnostic Radiology, Department of Diagnostic and Therapeutic Radiology, Faculty of Medicine Ramathibodi Hospital, Mahidol University, Bangkok, Thailand; ^3^Department of Clinical Epidemiology and Biostatistics, Faculty of Medicine Ramathibodi Hospital, Mahidol University, Bangkok, Thailand; ^4^Department of Medicine, Faculty of Medicine Ramathibodi Hospital, Mahidol University, Bangkok, Thailand; ^5^Medical and Health Department, Electricity Generating Authority of Thailand, Nonthaburi, Thailand

## Abstract

**Purpose:**

Osteoporotic VF is frequently asymptomatic and affects not only women but also men. Identifying patients at risk is essential for early management and prevention. BMD and the TBS are measurements of bone strength and trabecular microarchitecture, respectively. Their role in VF prediction in men is less well-studied. We determined the BMD and TBS predictive ability for osteoporotic VF in men.

**Methods:**

A total of 115 male participants of the Electricity Generating Authority of Thailand (EGAT) cohorts without a history of VF who completed the baseline BMD and TBS measurements in 2012 and a thoracolumbar spine radiograph in 2017 were recruited. The VF was assessed using the Genant semiquantitative method. Logistic regression analysis was performed to identify factors associated with the fracture. The area under the receiving operator curve (AUC) was analyzed to define VF predictive ability.

**Results:**

Forty subjects (34.78%) had VFs. The unadjusted relative risks (95% confidence interval) for VF for one standard deviation decrease in the TBS and low TBS were 1.319 (1.157–1.506) and 2.347 (1.496–3.682), respectively, and remained significant after BMD and age adjustment. For VF prediction, combined models had a greater AUC than models predicted from a single variable. The use of low TBS, femoral neck BMD, and age provided the best AUC (0.693).

**Conclusion:**

BMD and the TBS could predict osteoporotic VF in male EGAT employees. The use of both BMD and the TBS in the VF prediction process improved predictive ability.

## 1. Introduction

Osteoporosis is a disease characterized by low bone mass and microarchitectural deterioration of bone tissue, leading to enhanced bone fragility and increase risk for fragility or osteoporotic fracture [1, 2]. It is one of the important global health problems affecting not only women but also men. Most osteoporotic patients are asymptomatic and frequently present with osteoporotic fracture, commonly at the hip, spine, or wrist [3]. Approximately, 33% of the women and 20% of the men aged over 50 years will suffer osteoporotic fractures in their remaining lifetimes [4].

Vertebral fracture (VF) is the most common osteoporotic fracture but it frequently does not come to clinical attention, resulting in underdiagnosis [5]. Some of the patients subsequently display physical and mental illnesses, which reduce the quality of life, including chronic back pain, back deformity, respiratory dysfunction, and anxiety [6–8].

Bone mineral density (BMD), commonly measured by dual-energy X-ray absorptiometry (DXA), is used as an osteoporotic diagnostic tool [3]. Additionally, there is an indirect index of trabecular microarchitecture called the trabecular bone score (TBS), which is noninvasively evaluated by pixel gray-level variation in the two-dimensional lumbar spine DXA image. The TBS correlates with the strength of the trabecular microarchitecture and does not depend on BMD [9]. Adding the TBS to the fracture risk assessment tool (FRAX®) could further adjust the FRAX-probability of hip and major osteoporotic fractures in postmenopausal women and older men [10].

Several studies have shown that BMD and the TBS are associated with VF [11–17]. Each of the BMD and TBS can be used in VF prediction [13–17], and the combination of these two values further improves the prediction [13–15]. Most of these previous studies mainly focused on women [11, 13–15, 17], with only two of them studying men [12, 16].

This study aimed to determine the BMD and TBS predictive ability in osteoporotic VF in men.

## 2. Materials and Methods

### 2.1. Study Subjects

The source of the study population comprised male current and ex-employees at the headquarters of the Electricity Generating Authority of Thailand (EGAT), Bangkok, who participated in both the EGAT 1/5 and EGAT 1/6 cohort studies. The cohort profile has been described in a previous report [18]. This bone health study was extended from a cohort that was originally focused on cardiovascular risk factors.

**Figure 1:**
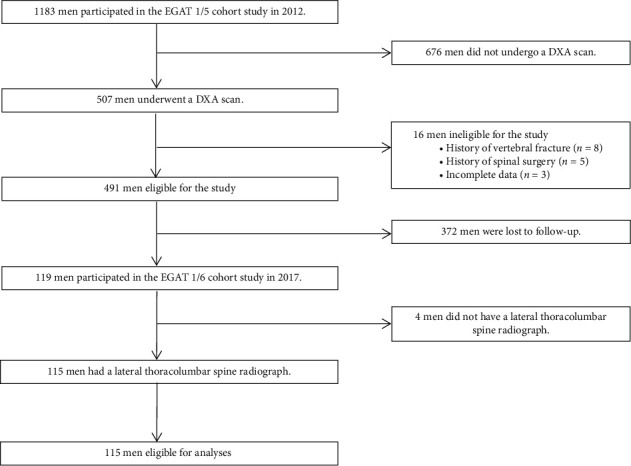
Flowchart of subject recruitment and follow-up.

A flowchart of subject recruitment and follow-up is presented in Figure 1. Of 1183 men participating in the EGAT 1/5 cohort study in 2012, 507 (42.86%.) underwent a DXA scan. Of 507 men, 16 were excluded because of their baseline information, 372 were lost to follow-up in the EGAT 1/6 cohort study in 2017, and 4 men were excluded because of unavailable lateral thoracolumbar spine radiograph, leaving a total of 115 men for the final analyses. None of them reported a history of high-energy fracture in the following cohort study in 2017.

The study was approved by the Institutional Review Board and by the Committee on Human Rights Related to Research Involving Human Subjects, Faculty of Medicine, Ramathibodi Hospital, Mahidol University. All subjects provided written informed consent before the commencement of the EGAT 1/5 and 1/6 cohort studies.

### 2.2. BMD Assessment

All subjects underwent BMD assessment at the lumbar spine (L1–L4 vertebrae) and hip (femoral neck (FN) and total hip (TH)). All measurement procedures operating in the fast array mode were performed by The International Society for Clinical Densitometry–certified densitometer technologists using the same Hologic Discovery W DXA scanner on all subjects (Hologic, Bedford, MA). Quality assurance procedures using a spine phantom were performed daily.

The lumbar spine (LS), TH, and FN T-scores were calculated using the mean and standard deviation (SD) of a female non-Hispanic white population aged 20–29 years from the National Health and Nutrition Examination Survey 2005–2008 [19]. According to WHO criteria [20], the T-score from each site and the lowest T-score were classified as normal (≥−1), low bone mass (between −1 and −2.5), or osteoporosis (≤−2.5).

### 2.3. TBS Assessment

The TBS was obtained from the baseline LS DXA image from 2012 using the TBS iNsight software version 2.1 (Medimaps, Mèrignac, France). A TBS ≤1.200 was considered degraded microarchitecture as previously proposed [9]. Using this cutoff, we classified participants into two groups: low TBS (≤1.200) and high TBS (>1.200).

### 2.4. Radiographic Assessment

In the EGAT 1/6 cohort study in 2017, lateral thoracolumbar spine radiographs were obtained from all participants.

After the nuclear medicine physician (CSa) was given VF assessment training in using the Genant semiquantitative method by the musculoskeletal radiologist (PF) [21], the agreement study between the nuclear medicine physician and musculoskeletal radiologist was performed by independently reviewing the radiographs of 34 subjects, as selected by systematic random sampling, in terms of the presence of a fracture. The agreement was 0.80, and the nuclear medicine physician subsequently reviewed all the spine radiographs to classify the participants into two groups: with or without VF. In the case of diagnostic uncertainty, consultation with the musculoskeletal radiologist was conducted.

### 2.5. Statistical Analysis

All statistical analyses were performed using STATA (version 16). A *p* value of 0.05 was set as the threshold for statistical significance. Continuous variables were reported as the mean with SD and categorical variables reported as number and percentage. Intergroup differences in variables between participants with and without VF were determined by student's *t*-test for continuous variables and Chi-square or Fisher's exact test for categorical variables. Univariate and multivariate logistic regression models were applied to evaluate predictors for VF expressing relative risk with a 95% confidence interval. Variables with statistical significance from the univariate analysis or clinical significance were included in the multivariate analysis. The ability of each fracture prediction model was determined by the area under the receiving operator curve (AUC) with a 95% confidence interval. AUCs were compared using DeLong test. Furthermore, the prevalence of osteoporotic VF classified by the levels of the TBS and LS T-score or lowest T-score groups were calculated.

## 3. Results

### 3.1. Description of the Study Subjects

This study included 115 male participants, 40 (34.78%) of whom had at least one VF at follow-up. Baseline characteristics of the participants with and without VF are presented in Table 1. The total mean height, weight, and body mass index (BMI) ± SD at baseline were 164.0 ± 4.9 cm, 65.8 ± 9.4 kg, and 24.4 ± 3.2 kg/m^2^, respectively. The total mean height, weight, and BMI ±SD at follow-up were 164.1 ± 5.0 cm, 64.8 ± 9.8 kg, and 24.0 ± 3.3 kg/m^2^, respectively. Overall, the participants had significantly lower weight and BMI at follow-up (both *p* < 0.05).

The mean baseline TBS, LS-BMD, TH-BMD, and FN-BMD ± SD of all participants were 1.303 ± 0.086, 0.929 ± 0.132 g/cm^2^, 0.911 ± 0.122 g/cm^2^, and 0.675 ± 0.092 g/cm^2^, respectively. Those with VF were older (*p*=0.016) and had a significantly lower TBS (*p*=0.001) and LS-BMD (*p*=0.0497) compared with those without VF. There were significantly more participants with a low TBS (*p*=0.002) in the VF group. No statistical differences in LS, FN, TH, and the lowest T-score groups were identified.

### 3.2. Prevalence of Vertebral Fracture

**Figure 2:**
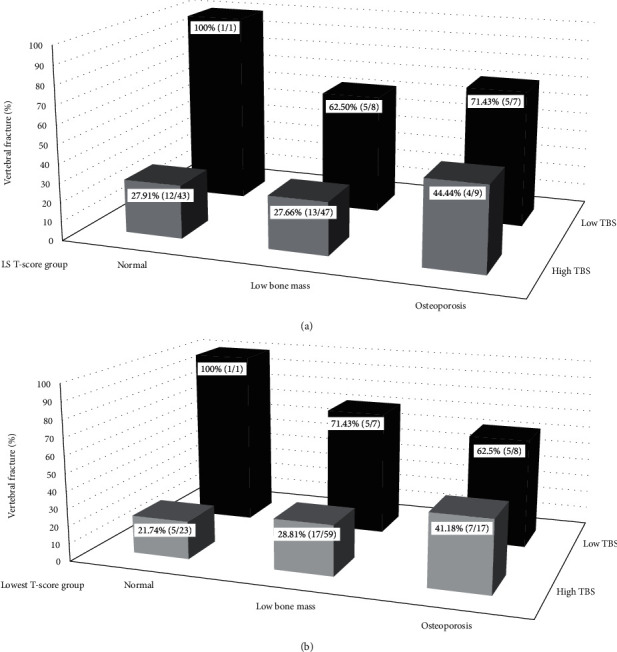
(a) Prevalence of vertebral fracture in participants classified by the levels of the trabecular bone score (TBS) and lumbar spine (LS) T-score groups. (b) Prevalence of vertebral fracture in participants classified by the levels of the TBS and lowest T-score groups.

The subjects were classified by the TBS levels (low and high TBS) and T-score groups (normal, low bone mass, and osteoporosis from the LS and the lowest T-score), and the prevalence was calculated as illustrated in Figure 2. A higher prevalence of VFs was found in the low TBS group compared with that in the high TBS group in all normal, low bone mass, and osteoporotic groups.

### 3.3. Logistic Regression Analyses

Univariate logistic regression analyses of all variables demonstrated that the age, TBS, low TBS, LS-BMD, and osteoporotic LS T-score were significantly associated with VF (Table 2). A one-year increase in age, one-SD decrease in the TBS and LS-BMD, and a low TBS were associated with an increase in the relative risk for VF (all *p* < 0.05). The osteoporotic LS T-score also increased the risk for VF compared with the normal LS T-score (*p*=0.045).

Multivariate logistic regression analysis revealed that when adjusting the TBS and low TBS by age and BMD at any site, both of the adjusted relative risks for VF remained significant (Table 3).

### 3.4. Vertebral Fracture Predictive Ability

Table 4 shows the predictive ability of models for VF that incorporated several variables as the AUC (95% CI). All combined models of the TBS or a low TBS, BMD of any site, and age had greater AUCs than models based on a single variable or both BMD of any site and age. However, the combined model of a low TBS, FN-BMD, and age, providing an AUC of 0.693, was the only model that significantly improved the AUC compared with a low TBS or FN-BMD alone (*p*=0.031). The AUC (95% confident interval) of the combined model of FN-BMD and a low TBS was 0.653 (0.545–0.760). Incorporating a low TBS and FN-BMD in the prediction significantly improved the AUC of the model based on FN-BMD (*p*=0.034). Incorporating the TBS or a low TBS into the combined models of BMD of any site and age did not significantly improve the AUCs.

## 4. Discussion

Osteoporosis is one of the most important health problems in both older females and males. It frequently results in osteoporotic fracture, which commonly occurs in the spine. Osteoporotic VF is frequently asymptomatic, leading to underdiagnosis and undertreatment [5]. Therefore, osteoporotic VF risk assessment is essential for early prevention and treatment. This retrospective cohort study examined 115 male EGAT employees with and without VF. The results revealed that both the TBS and BMD could predict osteoporotic VF.

The TBS, low TBS, and LS-BMD were significantly different between the participants with and without VF. In univariate analysis, a one-SD decrease in the LS-BMD was significantly associated with an increased risk for VF. This result is in agreement with previous studies in men [12] and women [11, 13–15, 17]. Age and the osteoporotic LS T-score were also significantly associated with an increased risk, similar to a previous study in men [12]. Neither the TH-BMD nor FN-BMD displayed a significant association with the VF in the present study, which is different from some studies [11–13] because of fewer and different subjects. For example, Legrand et al. only focused on men with an LS T-score below −1.5, whereas the present study included all classifications of the LS T-score. A one-SD decrease in the TBS significantly increased the VF risk, like previous studies in men [16] and women [13–15, 17]. A low TBS also increased the risk. This finding is comparable with the study in women 50 years of age or older by Hans et al. [13], which reported an increased odds ratio for major osteoporotic fracture in the lowest TBS tertile.

In multivariate analysis, we found a significantly increased risk for VF with a one-SD decrease in the TBS and a low TBS after adjusting for age and BMD at any site. Our results supported the previous study in elderly men [22], which reported that the TBS was associated with major osteoporotic fracture and could be used in conjunction with FRAX for fracture risk assessment. Among the all combined models for VF prediction, only the model of a low TBS, FN-BMD, and age displayed statistically significant improvement in the prediction compared with the model predicting from either a low TBS or FN-BMD and provided the best AUC in the present study. Adding a low TBS to the model consisting of FN-BMD and age did not demonstrate significantly increased AUC, probably due to the small number of subjects. However, using both FN-BMD and a low TBS significantly improved the prediction compared with using FN-BMD alone. This finding emphasized an additional benefit in VF risk stratification by incorporating a low TBS into the prediction model.

All three combined models of the TBS, BMD of any site, and age had greater AUC than single variable models, comparable with other studies in women [13–15], which reported that incorporating the TBS and BMD displayed better predictive performance than using either of these alone in the prediction. However, they did not show statistically significant improvement in the AUC, and their AUCs were less than the aforementioned studies shown in Table 5. Besides different sex, this was probably due to fewer subjects and shorter follow-up times in the present study.

A higher prevalence of VF among those with a low TBS was observed in all the groups classified by LS and the lowest T-score, demonstrating that the TBS was an independent risk factor for vertebral fracture. This result supported the previous studies in men which reported that increased TBS was associated with lower proportions of prevalent radiographic vertebral fracture [22], and there was greater loss of trabecular connectivity in osteoporotic men with fragility fracture than those without fragility fractures [23]. Unlike those with a high TBS, the prevalence of VF in the groups classified by LS and the lowest T-score (normal, low bone mass, and osteoporosis) with a low TBS did not increase in an orderly fashion due to the small number of subjects. For example, there was only one subject in the group of the low TBS with a normal LS T-score and the group of a low TBS and lowest T-score, and the subject also had a moderate degree of VF. Therefore, the calculated prevalence of VF was 100%.

This study had some limitations. First, the number of subjects was limited. There were 1183 men who participated in the cohort study in 2012, but only 507 completed the BMD assessment because there was limited time to perform a DXA scan. Due to elders' physical performance, it took a long time for subject positioning for the scan. Many of them were lost to follow-up in the cohort study in 2017, and some failed to obtain thoracolumbar spine radiographs, leaving a total of 115 men in this present study. Second, the radiograph quality was compromised in some regions of the thoracolumbar spine, possibly leading to outcome assessment bias. However, this affected only 3.3% of the thoracolumbar vertebrae. Finally, there was selection bias. This study examined male EGAT employees, representatives of the urban middle class, which was not a good representative sample of the Thai population. A further larger prospective study is required to confirm the predictive ability among the general Thai population.

## 5. Conclusions

BMD and the TBS could predict osteoporotic VF in male EGAT employees. The use of both BMD and the TBS in the VF prediction process improved the predictive ability.

## Figures and Tables

**Table 1:** Baseline characteristics of participants with and without vertebral fracture.

Characteristics	With vertebral fracture *n* = 40 (34.78%)	Without vertebral fracture *n* = 75 (65.22%)	*p* value
Age (years)	70.3 ± 4.8	68.2 ± 4.0	0.016
Height (cm)	163.6 ± 5.2	164.2 ± 4.6	0.510
Weight (kg)	67.1 ± 11.4	65.1 ± 8.1	0.276
BMI (kg/m^2^)	25.0 ± 3.7	24.1 ± 2.9	0.168
Current smoking *n* (%)	1 (100)	0	0.348
Current alcohol drinking *n* (%)	19 (35.85)	34 (64.15)	0.824
Diabetes mellitus *n* (%)	7 (24.14)	22 (75.86)	0.167
Hepatitis *n* (%)	0	6 (100)	0.09
TBS L1–L4	1.268 ± 0.088	1.321 ± 0.079	0.001
Low TBS (≤1.200) *n* (%)	11 (68.75)	5 (31.25)	0.002
LS-BMD (g/cm^2^)	0.896 ± 0.136	0.947 ± 0.127	0.0497
LS T-score			0.143
Normal *n* (%)	13 (29.55)	31 (70.45)	
Low bone mass *n* (%)	18 (32.72)	37 (67.27)	
Osteoporosis *n* (%)	9 (56.25)	7 (43.75)	
TH-BMD (g/cm^2^)	0.899 ± 0.109	0.917 ± 0.128	0.439
TH T-score			0.792
Normal *n* (%)	22 (32.84)	45 (67.16)	
Low bone mass *n* (%)	17 (36.96)	29 (63.04)	
Osteoporosis *n* (%)	1 (50)	1 (50)	
FN-BMD (g/cm^2^)	0.675 ± 0.092	0.715 ± 0.116	0.058
FN T-score			0.369
Normal *n* (%)	8 (27.59)	21 (72.41)	
Low bone mass *n* (%)	23 (34.33)	44 (65.67)	
Osteoporosis *n* (%)	9 (47.37)	10 (52.63)	
Lowest T-score			0.223
Normal *n* (%)	6 (25)	18 (75)	
Low bone mass *n* (%)	22 (33.33)	44 (66.67)	
Osteoporosis *n* (%)	12 (48)	13 (52)	

BMI, body mass index; TBS, trabecular bone score; BMD, bone mineral density; LS, lumbar spine; TH, total hip; FN, femoral neck.

**Table 2:** Univariate relative risk of vertebral fracture prediction.

Predictor	RR (95% CI)	*p* value
Age (1 year increase)	1.064 (1.013–1.117)	0.013
Height (1 cm increase)	0.980 (0.931–1.031)	0.437
Weight (1 kg increase)	1.020 (0.994–1.047)	0.134
BMI (1 kg/m^2^ increase)	1.075 (0.997–1.160)	0.061
TBS L1–L4 (1 SD decrease)	1.319 (1.157–1.506)	<0.01
Low TBS (≤1.200)	2.347 (1.496–3.682)	<0.01
LS-BMD (1 SD decrease)	1.314 (1.020–1.692)	0.034

*LS T-score*		
Normal	Reference	
Low bone mass	1.108 (0.0612–2.004)	0.735
Osteoporosis	1.904 (1.016–3.569)	0.045
TH-BMD (1 SD decrease)	1.100 (0.856–1.414)	0.455

*TH T-score*		
Normal	Reference	
Low bone mass	1.125 (0.6761–1.874)	0.649
Osteoporosis	1.523 (0.365–6.348)	0.564
FN-BMD (1 SD decrease)	1.287 (0.986–1.681)	0.063

*FN T-score*		
Normal	Reference	
Low bone mass	1.244 (0.633–2.447)	0.526
Osteoporosis	1.717 (0.806–3.659)	0.161

*Lowest T-score*		
Normal	Reference	
Low bone mass	1.333 (0.616–2.887)	0.465
Osteoporosis	1.92 (0.859–4.291)	0.112

RR, relative risk; CI, confidence interval; SD, standard deviation; BMI, body mass index; TBS, trabecular bone score; BMD, bone mineral density; LS, lumbar spine; TH, total hip; FN, femoral neck.

**Table 3:** Multivariate-adjusted relative risk of vertebral fracture for one-SD decrease in TBS and BMD, one-year increase in age.

	Predictor	Adjusted RR (95% CI)	*p* value
1	TBS	1.304 (1.074–1.582)	0.007
	LS-BMD	1.028 (0.802–1.318)	0.829
	Age	1.053(1.004–1.104)	0.033
2	TBS	1.385 (1.120–1.712)	0.003
	TH-BMD	0.894 (0.665–1.202)	0.458
	Age	1.054 (1.010–1.099)	0.016
3	TBS	1.264 (1.048–1.527)	0.014
	FN-BMD	1.127 (0.813–1.567)	0.476
	Age	1.056 (1.008–1.107)	0.023
4	Low TBS	2.081 (1.058–4.093)	0.034
	LS-BMD	0.985 (0.743–1.309)	0.920
	Age	1.051 (0.998–1.106)	0.058
5	Low TBS	2.114 (1.284–3.480)	0.003
	TH-BMD	0.944 (0.716–1.244)	0.682
	Age	1.052 (1.001–1.106)	0.045
6	Low TBS	1.851 (1.136–3.017)	0.013
	FN-BMD	1.163 (0.847–1.597)	0.351
	Age	1.048 (0.998–1.101)	0.061

RR, relative risk; CI, confidence interval; SD, standard deviation; TBS, trabecular bone score; BMD, bone mineral density; LS, lumbar spine; TH, total hip; FN, femoral neck.

**Table 4:** AUC (95% CI) for the prediction of vertebral fracture for one-SD decrease in the TBS and BMD and one-year increase in age.

	Predictor models	AUC (95% CI)
Univariate	TBS	0.667 (0.562–0.772)
	Low TBS	0.604 (0.529–0.680)
	LS-BMD	0.609 (0.497–0.722)
	TH-BMD	0.524 (0.411–0.637)
	FN-BMD	0.588 (0.479–0.696)
Combined	LS-BMD, age	0.657 (0.547–0.767)
	TH-BMD, age	0.629 (0.516–0.741)
	FN-BMD, age	0.561 (0.544–0.759)
Combined	TBS, LS-BMD, age	0.686 (0.584–0.789)
	TBS, TH-BMD, age	0.688 (0.585–0.791)
	TBS, FN-BMD, age	0.690 (0.588–0.792)
Combined	Low TBS, LS-BMD, age	0.665 (0.550–0.779)
	Low TBS, TH-BMD, age	0.672 (0.559–0.786)
	Low TBS, FN-BMD, age	0.693 (0.588–0.798)^*∗*^

AUC, area under the receiving operator curve; CI, confidence interval; SD, standard deviation; TBS, trabecular bone score; BMD, bone mineral density mineral density; LS, lumbar spine; TH, total hip; FN, femoral neck. ^*∗*^Statistically significant improvement in the AUC compared with the model predicting from either a low TBS or FN-BMD.

**Table 5:** AUC (95% CI) for predicting vertebral fracture in the present study compared with two previous studies [13, 14].

AUC (95% CI)	This study *n* = 115 retrospective cohort	Hans et al. [13] *n* = 29,407 retrospective cohort	Iki et al. [14] *n* = 665 prospective cohort
TBS, LS-BMD	0.686^a^ (0.584–0.789)	0.71 (0.69–0.74)	0.718^a^ (0.662–0.773)
TBS, TH-BMD	0.688^a^ (0.585–0.791)	0.73 (0.71–0.75)	—
TBS, FN-BMD	0.690^a^ (0.588–0.792)	0.73 (0.71–0.75)	—

*n*, study subjects; AUC, area under the receiving operator curve; CI, confidence interval; TBS, trabecular bone score; BMD, bone mineral density; LS, lumbar spine; TH, total hip; FN, femoral neck. ^a^Combined with age.

## Data Availability

The data used to support the results of this study are available from the corresponding author upon request.
